# Diagnostic ability of artificial intelligence using deep learning analysis of cyst fluid in differentiating malignant from benign pancreatic cystic lesions

**DOI:** 10.1038/s41598-019-43314-3

**Published:** 2019-05-03

**Authors:** Yusuke Kurita, Takamichi Kuwahara, Kazuo Hara, Nobumasa Mizuno, Nozomi Okuno, Shimpei Matsumoto, Masahiro Obata, Hiroki Koda, Masahiro Tajika, Yasuhiro Shimizu, Atsushi Nakajima, Kensuke Kubota, Yasumasa Niwa

**Affiliations:** 1 Department of Gastroenterology, Aichi Cancer Center Hospital, Nagoya, Japan; 2 Department of Endoscopy, Aichi Cancer Center Hospital, Nagoya, Japan; 3 Department of Gastroenterological Surgery, Aichi Cancer Center Hospital, Nagoya, Japan; 4 Department of Gastroenterology and Hepatology, Yokohama City University School of Medicine, Yokohama, Japan

**Keywords:** Gastrointestinal cancer, Pancreatic disease, Pancreas

## Abstract

The diagnosis of pancreatic cystic lesions remains challenging. This study aimed to investigate the diagnostic ability of carcinoembryonic antigen (CEA), cytology, and artificial intelligence (AI) by deep learning using cyst fluid in differentiating malignant from benign cystic lesions. We retrospectively reviewed 85 patients who underwent pancreatic cyst fluid analysis of surgical specimens or endoscopic ultrasound-guided fine-needle aspiration specimens. AI using deep learning was used to construct a diagnostic algorithm. CEA, carbohydrate antigen 19-9, carbohydrate antigen 125, amylase in the cyst fluid, sex, cyst location, connection of the pancreatic duct and cyst, type of cyst, and cytology were keyed into the AI algorithm, and the malignant predictive value of the output was calculated. Area under receiver-operating characteristics curves for the diagnostic ability of malignant cystic lesions were 0.719 (CEA), 0.739 (cytology), and 0.966 (AI). In the diagnostic ability of malignant cystic lesions, sensitivity, specificity, and accuracy of AI were 95.7%, 91.9%, and 92.9%, respectively. AI sensitivity was higher than that of CEA (60.9%, p = 0.021) and cytology (47.8%, p = 0.001). AI accuracy was also higher than CEA (71.8%, p < 0.001) and cytology (85.9%, p = 0.210). AI may improve the diagnostic ability in differentiating malignant from benign pancreatic cystic lesions.

## Introduction

There are different types of pancreatic cystic lesions, such as intraductal papillary mucinous neoplasm (IPMN), mucinous cystic neoplasm (MCN), serous cystic neoplasm (SCN), and pancreatic pseudocyst (PPC). In IPMN, high-grade dysplasia or invasive carcinoma is often observed in 43% to 62% of patients who undergo surgical resection^1^. IPMN with high-grade dysplasia or invasive carcinoma should be resected surgically, while IPMN with low- or intermediate-grade dysplasia should be monitored^2^. In MCN, invasive carcinoma is recognized in 4% to 16% of surgical resections^3–5^, and current guidelines recommend surgical resection^1^. On the contrary, SCN and PPC are reported to have a slight risk of malignancy^6^. Therefore, it is important to differentiate malignant from benign pancreatic cystic lesions to determine the appropriate treatment strategy. However, it is difficult to differentiate malignant from benign pancreatic cystic lesions including SCN, PPC, epidermoid cysts (EDC), and lymphoepithelial cysts (LEC) based on clinical presentation and imaging modalities, using computed tomography (CT) and endoscopic ultrasound (EUS)^7–9^. Pancreatic cyst fluid analysis of several tumour markers, including carcinoembryonic antigen (CEA), carbohydrate antigen (CA) 72-4, CA125, CA19-9, and CA15-3, has been evaluated to determine whether the lesion is mucinous^10^. The CEA of the pancreatic cyst fluid has been the most helpful tumour marker in differentiating mucinous from non-mucinous pancreatic cystic lesions^11–13^. Conversely, cyst fluid CEA level including other tumour markers is not useful in differentiating malignant from benign cystic lesions^12,14–16^. Clinical data of pancreatic cysts have been reported variously in terms of sex^17,18^, location of the cystic lesion (e.g., MCN is commonly located in tails)^5^, connection of the pancreatic duct and cyst^1,5,19^, type of cyst (monolocular or multilocular, cysts excluding pseudocysts are common in multilocular cysts)^20^, but even if these clinical features are used, it is also difficult to diagnose the malignancy of pancreatic cysts. Therefore, diagnosis in differentiating malignant from benign pancreatic cystic lesions is challenging.

An artificial neural network that imitates the cranial nervous system is a type of machine learning system, that is, artificial intelligence (AI). An artificial neural network consists of an input layer, a hidden layer, and an output layer. An artificial neural network with multiple hidden layers is called deep learning^21^, which aims at learning multilevel representations of data to make predictions or classifications. This AI uses deep learning to analyse various images and extract clinical data using specific algorithm. AI using deep learning has been used and validated in various medical fields^22–27^.

Therefore, this study aimed to investigate and compare the diagnostic ability of cyst fluid analysis of tumour markers and amylase, cytology, and AI combining pancreatic cyst fluid analysis and clinical data in differentiating malignant from benign pancreatic cystic lesions.

## Results

A total of 138 patients underwent pancreatic cystic lesions analysis, of which 53 patients with missing data were excluded. Eighty-five patients were analysed in this study. The mean age was 58.2 ± 13.4 years, 35 were men, and 50 were women (Table 1). The final diagnosis of 23 patients was malignant and that of 62 patients was benign. Specimens were obtained by surgical method in 59 patients and by ultrasound-guided fine needle aspiration (EUS-FNA) in 26 patients. The median age (p = 0.003), location of the cystic lesion (p = 0.031), connection of the main pancreatic duct and cyst (p < 0.001), CEA (p = 0.002), and malignant predictive value by AI (p < 0.001) and malignant predictive value by AI using only CEA (p < 0.001) of malignant cystic lesions were significantly different compared to that of benign lesions, but no significant difference was found between malignant and benign cystic lesions in terms of sex, type of cyst, CA19-9, CA125, and amylase. Thirty patients had IPMN, among which 19 had malignancy. Twenty-three patients had MCN, of which four had malignancy (Table 2).

**Table 1:** Baseline characteristics.

Variable	N = 85	Benign	Malignant	p
Patient		n = 62	n = 23	
Age (years), mean ± SD	58.2 ± 13.4	57.0 ± 13.7	65.4 ± 9.8	0.003^a^
Sex (%)				
Male	35 (41.2)	22 (35.5)	13 (56.5)	0.080^a^
Female	50 (58.8)	40 (64.5)	10 (43.5)	
Location of cystic lesion (%)				
Head	29 (34.1)	20 (32.3)	9 (39.1)	0.031^a^
Body	25 (29.4)	23 (37.1)	2 (8.7)	
Tail	31 (36.5)	19 (30.6)	12 (52.2)	
Connection of main pancreatic duct and cyst (%)				
Present	34 (40.0)	16 (25.8)	18 (78.3)	<0.001^a^
Absent	51 (60.0)	46 (74.2)	5 (21.7)	
Type of cyst (%)				
Monolocular	16 (18.8)	15 (24.2)	1 (4.3)	0.058^b^
Multilocular	69 (81.2)	47 (75.8)	22 (95.7)	
CEA (ng/mL), median (IQR)	243.6 (17.0–8325.3)	132.3 (4.8–1232.2)	1407.1 (111.5–30300.0)	0.002^c^
CA19-9 (LU/mL), median (IQR)	4740.0 (411.6–357855.0)	3273.0 (305.4–346927.5)	33180.0 (1018.0–550000.0)	0.306^c^
CA125 (U/mL), median (IQR)	45.5 (6.5–1300.3)	52.5 (9.8–1435.8)	33.0 (4.0–1070.0)	0.406^c^
Amylase (U/L), median (IQR)	3101.0 (112.0–25917.5)	2934.0 (116.8–38837.8)	3430.0 (100.0–11892.0)	0.318^c^
Cyst fluid sampling procedure (%)				
Surgery specimen	59 (69.4)	38 (61.3)	21 (91.3)	0.008^a^
EUS-FNA	26 (30.6)	24 (38.7)	2 (8.7)	
Malignant predictive value by AI, median (range)	0.068 (0.00–0.99)	0.049 (0.00–0.79)	0.928 (0.05–0.99)	<0.001^c^
Malignant predictive value by AI using only CEA, median (range)	0.101 (0.01–0.97)	0.072 (0.01–0.80)	0.902 (0.07–0.97)	<0.001^c^

CEA, carcinoembryonic antigen; CA19-9, carbohydrate antigen 19-9; CA125, carbohydrate antigen 125; EUS-FNA, endoscopic ultrasound-guided fine needle aspiration; IQR, interquartile range; SD, standard deviation.

p^a^ Chi-squared test, p^b^ Fisher’s exact test, p^c^ Mann-Whitney U test.

**Table 2:** Final diagnosis of pancreatic cystic lesions.

Number	N = 85
IPMN	30
Low- or intermediate-grade dysplasia	11
High-grade dysplasia	7
Invasive carcinoma	12
MCN	23
Low- or intermediate-grade dysplasia	19
High-grade dysplasia	2
Invasive carcinoma	2
SCN	15
PPC	13
EDC	2
LEC	2

EDC, epidermoid cyst; IPMN, intraductal papillary mucinous neoplasm; LEC, lymphoepithelial cyst; MCN, mucinous cystic neoplasm; PPC, pancreatic pseudocyst; SCN, serous cystic neoplasm.

### Diagnostic ability of CEA, CA19-9, CA125, and amylase using cyst fluid

**Figure 1:**
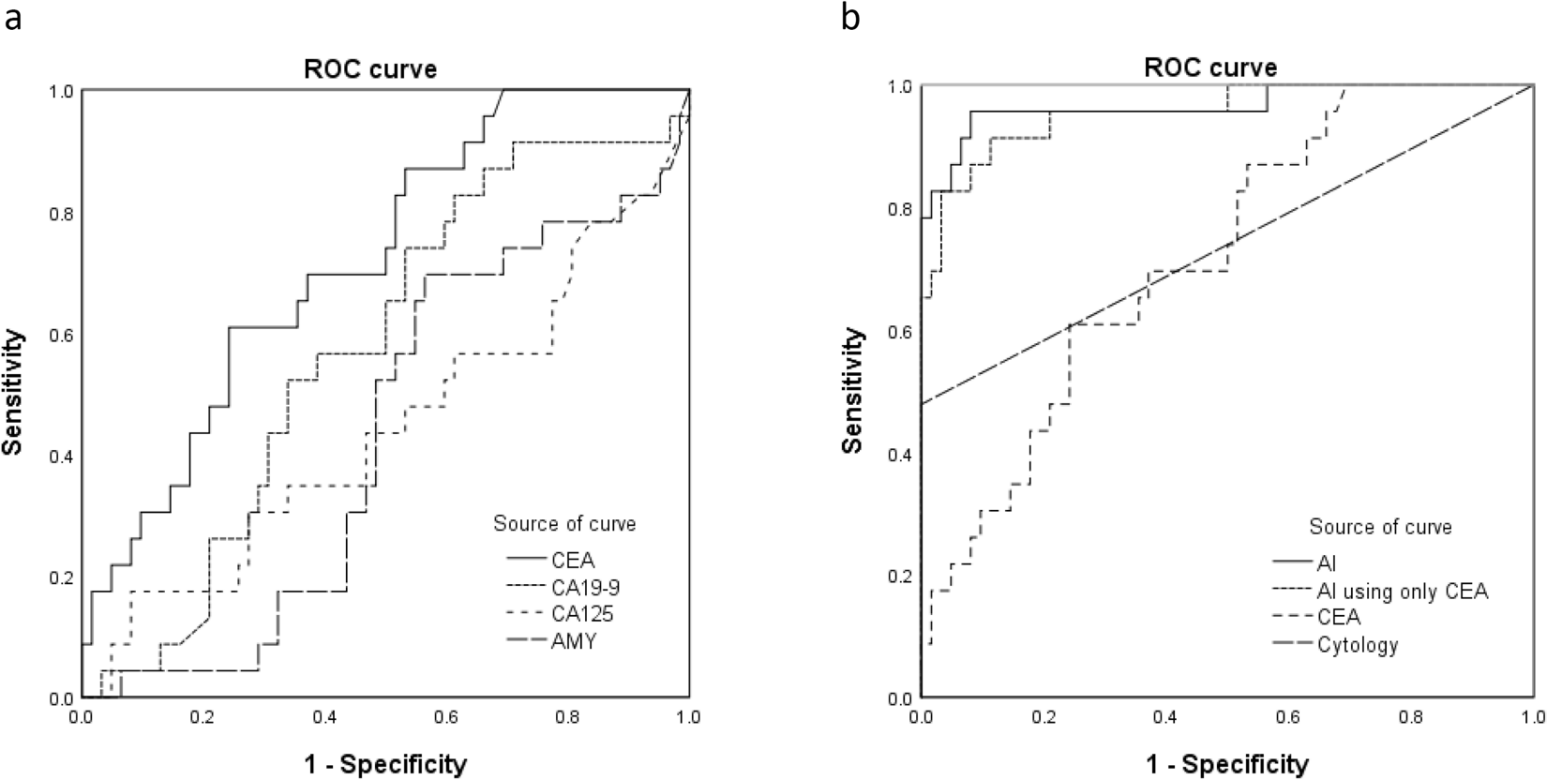
(**a**) Receiver-operating characteristics (ROC) curves for the tumour markers and amylase levels of cyst fluid in differentiating malignant from benign cystic lesions. Areas under the ROC curve (AUC) were as follows: carcinoembryonic antigen (CEA), 0.719; carbohydrate antigen (CA) 19-9, 0.573; CA125, 0.441; amylase, 0.429. (**b**) ROC curves for artificial intelligence (AI), AI using only CEA, CEA, and cytology of cyst fluid in differentiating malignant from benign cystic lesions. Areas under the ROC curves were as follows: AI, 0.966; AI using only CEA, 0.956; CEA, 0.719; cytology, 0.739.

The area under (AUC) a receiver operating characteristic (ROC) curve for diagnostic ability in differentiating malignant from benign pancreatic cystic lesions were as follows: CEA, 0.719 (p = 0.002); CA19-9, 0.573 (p = 0.306); CA125, 0.441 (p = 0.406), and amylase, 0.429 (p = 0.318). Among them, the AUC of CEA was the highest and significant variable. The ROC curve analysis of CEA, CA19-9, CA125, and amylase demonstrated (Fig. 1a) the AUC of the diagnostic ability in differentiating malignant from benign pancreatic cystic lesions. In this data, the cut-off CEA level of the highest diagnostic ability was 1154.7 ng/mL. At this cut-off level, the diagnostic ability of CEA in differentiating malignant from benign cystic lesions was confirmed with the following values: sensitivity, 60.9% (14/23); specificity, 75.8% (47/62); positive predictive value (PPV), 48.3% (14/29); negative predictive value (NPV), 83.9% (47/56); and accuracy, 71.8% (61/85).

### Diagnostic ability of cyst fluid cytology

The diagnostic ability of cytology was shown with following values: sensitivity, 47.8% (11/23); specificity, 100% (62/62); PPV, 100% (11/11); NPV, 83.8% (62/74); and accuracy, 85.9% (73/85).

### Diagnostic ability of AI using deep learning

The median malignant predictive value by AI of malignant cystic lesions was significantly higher than that of benign cystic lesions (0.928 vs. 0.049, p < 0.001). The ROC curve analysis of the malignant predictive value by AI was demonstrated, and the cut-off malignant predictive value by AI level of the highest diagnostic ability in differentiating malignant from benign pancreatic cystic lesions was 0.212. The diagnostic ability of AI was as follows: sensitivity, 95.7% (22/23); specificity, 91.9% (57/62); PPV, 81.5% (22/27); NPV 98.3% (57/58); and accuracy, 92.9% (79/85) respectively. The median malignant predictive value by AI using only CEA of malignant cystic lesions was significantly higher than that of benign cystic lesions (0.902 vs. 0.072, p < 0.001). The cut-off malignant predictive value by AI using only CEA level of the highest diagnostic ability in differentiating malignant from benign pancreatic cystic lesions was 0.238. The diagnostic ability of AI using only CEA was as follows: sensitivity, 91.3% (21/23); specificity, 88.7% (55/62); PPV, 75.0% (21/28); NPV 96.5% (55/57); and accuracy, 89.4% (76/85).

### Comparison of diagnostic ability of cyst fluid analysis, cytology, and AI

Comparison results of the diagnostic abilities of AI, AI using only CEA, cyst fluid analysis (CEA), and cytology in differentiating malignant from benign pancreatic cystic lesions is shown in Table 3. ROC curve analysis demonstrated (Fig. 1b) that the AUC of the diagnostic ability in differentiating malignant from benign pancreatic cystic lesions were as follows: AI, 0.966 (p < 0.001); AI using only CEA, 0.956 (p < 0.001); CEA, 0.719 (p = 0.002); and cytology, 0.739 (p = 0.001). Among them, AI has the highest AUC. The sensitivity of AI (95.7%) was higher than that of AI using only CEA (91.3%, p = 1.000), CEA (60.9%, p = 0.021), and cytology (47.8%, p = 0.001). The accuracy of AI (92.9%) was also higher than those of AI using only CEA (89.4%, p = 0.375), CEA (71.8%, p < 0.001), and cytology (85.9%, p = 0.210). The univariate and multivariate analyses of malignant cystic lesion including AI, pancreatic cyst fluid, and clinical data are shown in Table 4. In the univariate analysis, CEA, connection of the pancreatic duct and cyst, and AI were statistically significant. Variables with p < 0.05 in the univariate analysis were selected for entry into the multivariate analysis. In the multivariate analysis, CEA (p = 0.036; odds ratio [OR], 31.5; 95% confidence interval [CI], 1.3–794.4) and AI (p < 0.001; OR, 177.2; 95% CI, 12.0–2612.2) were significant factors in the diagnosis of malignant cysts.

**Table 3:** Diagnostic ability of cyst fluid analysis, cytology, and AI in differentiating malignant and benign lesions.

	AI	AI using only CEA	CEA	Cytology	p*	p^†^	p**
Sensitivity	95.7% (22/23)	91.3% (21/23)	60.9% (14/23)	47.8% (11/23)	1.000	0.021	0.001
Specificity	91.9% (57/62)	88.7% (55/62)	75.8% (47/62)	100.0% (62/62)	0.625	0.031	<0.001
PPV	81.5% (22/27)	75.0% (21/28)	48.3% (14/29)	100.0% (11/11)	—	—	—
NPV	98.3% (57/58)	96.5% (55/57)	83.9% (47/56)	83.8% (62/74)	—	—	—
Accuracy	92.9% (79/85)	89.4% (76/85)	71.8% (61/85)	85.9% (73/85)	0.375	<0.001	0.210
AUC	0.966	0.956	0.719	0.739			

AI, artificial intelligence; AUC, area under the receiver-operating characteristics curve; CEA, carcinoembryonic antigen; CA19-9, carbohydrate antigen 19-9; CA125, carbohydrate antigen 125; NPV, negative predictive value; PPV, positive predictive value.

p* McNemar test; AI vs AI using only CEA.

p^†^ McNemar test; AI vs CEA.

p** McNemar test; AI vs cytology.

**Table 4:** Univariate and multivariate analyses of malignant cystic lesion.

Variable		Number	Univariate	Multivariate	OR	95% CI
			p	p		
CEA	Positive	29	0.002	0.036	31.5	1.3–794.4
	Negative	56				
CA19-9	Positive	57	0.071			
	Negative	28				
CA125	Positive	64	0.194			
	Negative	21				
Amylase	Positive	20	0.065			
	Negative	65				
Sex	Male	35	0.084			
	Female	50				
Location of cystic lesion	Head	29	0.476			
	Body	25				
	Tail	31				
Connection of pancreatic	Present	34	<0.001	0.103	12.8	0.6–273.4
duct and cyst	Absent	51				
Type of cyst	Multilocular	69	0.067			
	Monolocular	16				
Cytology	Positive	11	0.998			
	Negative	74				
AI	Positive	27	<0.001	<0.001	177.2	12.0–2612.2
	Negative	58				

AI, artificial intelligence; CEA, carcinoembryonic antigen; CA19-9, carbohydrate antigen 19-9; CA125, carbohydrate antigen 125; CI, confidence interval; OR, odds ratio.

## Discussion

Diagnosis of malignant pancreatic cystic lesions is necessary for determining the appropriate treatment strategy. The diagnostic ability of CEA using pancreatic cyst fluid for malignant cysts was poor, and a previous meta-analysis reported its diagnostic sensitivity of 63% and specificity of 63%^14^. Even in the present study, the diagnostic ability of CEA by cyst fluid analysis was poor. Several studies have noted elevated cyst fluid CEA in LEC^28^ and EDC^29^. These studies reported CEA expression on the surface of squamous epithelial cells, indicating that squamous epithelial cells could produce CEA, resulting in the elevated cyst fluid CEA levels. In our study, two patients had EDC and two patients had LEC. These results increased the cut-off value of CEA in differentiating malignant from benign pancreatic cystic lesions. This is an important limitation of differentiating malignant from benign cystic lesions using cyst fluid CEA.

Although cytology had excellent specificity, it has a limited role because of its lack of sensitivity in previous studies^30–32^. In the present study, the sensitivity of cytology in differentiating malignant from benign cystic lesions was 47.8%. Thus, we constructed AI using deep learning algorithm for differentiating malignant from benign pancreatic cystic lesions based on the analysis of pancreatic cyst fluid and clinical data.

AI using deep learning is used for various fields such as skin cancer^22^, radiation oncology^23^, *Helicobacter pylori* infection^24^, colorectal polyp^25,26^, and histopathology^27^ using image information. However, the application of AI using deep learning to analyse pancreatic cystic lesions is relatively new. We used a system of AI using deep learning to simulate the human brain nervous system. In this study, we constructed AI using deep learning with TensorFlow. This framework accepts sets of data and corresponding labels as input clinical data and constructs a neural network for diagnosis. In this study, AI using deep learning analysed pancreatic cyst fluid and clinical data. By using this deep learning method, AI learns the characteristics of malignant cystic lesions by combining cyst fluid analysis and clinical data, and AI can possibly exclude the bias generated by human judgment. Although it is difficult for clinicians to diagnose malignant pancreatic cystic lesions by cyst fluid analysis and clinical data, AI using deep learning achieved adequate diagnostic ability in differentiating malignant from benign cystic lesions compared to cyst fluid analysis such as CEA and cytology. AI and CEA were also significant factor in the multivariate analysis of malignant cystic lesion. Specifically, although it is generally a problem that cytology diagnosis has low sensitivity, AI using deep learning achieved high sensitivity (95.7%). AI could be a powerful tool for exclusion diagnosis of malignant pancreatic cystic lesions. Although AI using only CEA was slightly lower in diagnostic ability than AI, AI using only CEA has good diagnostic ability. If CA 19-9, CA 125, and amylase cannot be used in the cystic fluid analysis, AI using only CEA may be useful for diagnosis in differentiating malignant from benign pancreatic cystic lesions. AI will improve the diagnostic ability of pancreatic cystic lesions if introduced as a supporting system. In the future, an AI using deep learning-based diagnostic system will change the method of diagnosis of differentiating malignant from benign pancreatic cystic lesions.

This study has some limitations. First, it is a retrospective and single-centre study. Second, this study had a small sample size and many cases were excluded (53/138 cases). The study is as long as 20 years, and we excluded cases with incorrect data to maintain the accuracy of the study. The AI algorithm cannot analyse data when there is a missing item. Hence, by improving the system and AI performance through developing new algorithms, it is desirable to achieve the good diagnostic ability of AI even if some items are missing. In addition, Because of the small sample size, we used cross-validation method, and verification with external data was not done. Hence, the diagnostic ability of AI using external data should be confirmed in the future. Thrid, in this study, subjective factors such as connection of the main pancreatic duct and cyst and type of cyst (monolocular or multilocular) were adopted. It is desirable to create algorithms that can be analysed in the future using only objective data, such as tumour marker of the pancreatic cyst fluid, age, and sex. Fourth, 30.6% (26/85) of cases using pancreatic cyst fluid was collected by EUS-FNA in this study. Analysis and cytology by EUS-FNA for pancreatic cyst fluid are widely used worldwide. Previous studies reported that the frequency of peritoneal seeding in the EUS-FNA group and the no sampling group has no significant difference^33^. However, EUS-FNA for the diagnosis of mucinous cystic lesions is rarely performed in Japan because there was one report of fatal peritoneal tumour seeding following EUS-FNA in Japan^34^. Therefore, there are only a few cases of pancreatic cyst fluid collection by EUS-FNA, especially cases suspected of mucinous cystic lesion prior to fluid analysis. A study reported that the rate of obtaining adequate amount of specimen through EUS-FNA was low and may not have sufficient diagnostic ability^35^. The diagnostic ability of cytology between surgical specimens and EUS-FNA may differ; thus, interpretation of the results of cytology in this study requires caution. Further, cytology was considered an effective factor for the AI algorithm, so cytology was used for the AI algorithm in this study. If EUS-FNA cytology is positive, AI may not be needed. In addition, developing an AI algorithm that excludes cytology is expected in the future.

In conclusion, AI may improve the diagnostic ability of differentiating malignant from benign cystic lesions. Moreover, compared to pancreatic cyst fluid analysis such as CEA and cytology, AI is highly sensitive of differentiating malignant from benign cystic lesions and may be useful for exclusion of malignant pancreatic cystic lesions.

## Methods

### Patients

We retrospectively reviewed data of patients with pancreatic cystic lesions who underwent cyst fluid analysis and cytology with surgical specimens or EUS-FNA from January 1997 to December 2017 in Aichi Cancer Center hospital. Patients who underwent cyst fluid analysis (CEA, CA19-9, CA125, amylase, and cytology) were included in this study, and patients with missing data were excluded. The cysts were IPMN, MCN, SCN, PPC, EDC, and LEC. Clinical data, biochemical, and pathological results were collected. To analyse AI algorithm, sex, location of the cystic lesion, connection of the pancreatic duct and cyst, type of cyst (monolocular or multilocular) were extracted as characteristic factors of pancreatic cysts. In this study, image data were referenced to results of CT, magnetic resonance cholangiopancreatography (MRCP), and EUS. CT, MRCP, and EUS images were reviewed by an experienced pancreatic radiologist and endoscopist.

This study was approved by the institutional review board of Aichi Cancer Center Hospital (No.2018-1-022). In this retrospective observational study, only medical information without invasion to participants is used. Informed consent for all participants was acquired in the form of opt-out. Those who rejected were excluded. All research and examinations were performed in accordance with relevant guidelines and regulations. All authors had agreed to all the contents and were involved in the review and approval of the manuscript.

### Pancreatic cyst fluid sampling method

Pancreatic cyst fluid was collected surgically or by EUS-FNA. Surgically, a cyst of a resected specimen was punctured with a needle and manually aspirated, and cyst fluid was collected. EUS-FNA procedures were performed with the patient under conscious sedation using 35 mg of intravenous pethidine hydrochloride (Mitsubishi Tanabe Pharma, Osaka, Japan) and 5–10 mg of intravenous midazolam (Astellas, Tokyo, Japan). EUS-FNA was performed using GF-UC30P, GF-UC240P-AL5, GF-UCT240-AL5, and GF-UCT260-AL5 convex linear echoendoscope (Olympus Medical Systems, Tokyo, Japan) connected to an ultrasound scanning system (SSD-5500, Prosound SSD a-10; Hitachi Aloka Medical, Tokyo Japan or EU-ME2; Olympus Medical Systems, Tokyo, Japan). Different needle types (19-, 22-, or 25-gauge Echo Tip Ultra; Cook Medical, IN, USA, or NA-U200H; Olympus Medical Systems, Tokyo, Japan, or Expect; Boston Scientific, Tokyo, Japan) were employed. Needle sizes and types were chosen at the discretion of the endosonographer. We uniformly used negative suction with a 10-mL or 20-mL syringe during all FNA procedures.

### Final diagnosis and definition of malignant and benign cystic lesions

**Figure 2:**
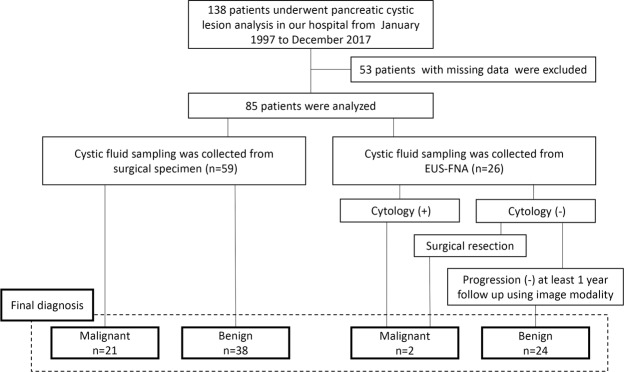
Flow chart leading to the final diagnosis.

Histopathological examination of the surgical specimen was regarded as the final diagnosis in case of surgical resection. For patients who did not undergo surgery, they were clinically diagnosed by clinical data, image diagnosis, and follow-up for 1 year or more. In particular, diagnosis of benign lesions was made in patients in whom we collected pancreatic cyst fluid by EUS-FNA when there were no malignant findings on cytology of the EUS-FNA and imaging examinations (CT, MRCP, and EUS reviewed by radiologist and endoscopist) and a lack of progression for 1 year or more on follow-up CT, MRCP, and EUS. On the contrary, in patients in whom pancreatic cyst was collected by EUS-FNA, malignant lesions were defined as lesions when surgical resection at a later date was malignant or pancreatic cyst fluid cytology was malignant. Figure 2 summarises the routes by which the 85 patients were finally diagnosed.

For malignant and benign classifications of cystic lesions, high-grade dysplasia or invasive carcinoma (IPMN or MCN) was considered a malignancy, and low- or intermediate-grade dysplasia (IPMN or MCN), PPC, EDC, and LEC were considered benign lesions.

### Pancreatic cyst fluid analysis and cytology

Pancreatic cyst fluid was taken for analysis of CEA, CA19-9, CA125, and amylase^10^. Cyst fluid concentrations of CEA, CA19-9, CA125, and amylase were measured by specific chemiluminescent immunoassays. CEA was analysed on ARCHITECT analyser (Abbot Japan, Tokyo, Japan). CA19-9 and CA125 were analysed on LUMIPULSE (FUJIREBIO Inc., Tokyo, Japan). Amylase was analysed on LABOSPECT 008 (Hitachi High-technologies Corporation, Tokyo, Japan). Pancreatic cyst fluid was also taken for cytology. Results of cytology were classified as benign, atypical, suspicious for malignancy, consistent with malignancy, and malignancy. Cytology was considered malignant when the report was suspicious for malignancy, consistent with malignancy, or malignancy. Benign and atypical in cytology were considered benign.

### Artificial intelligence using deep learning algorithm

**Figure 3:**
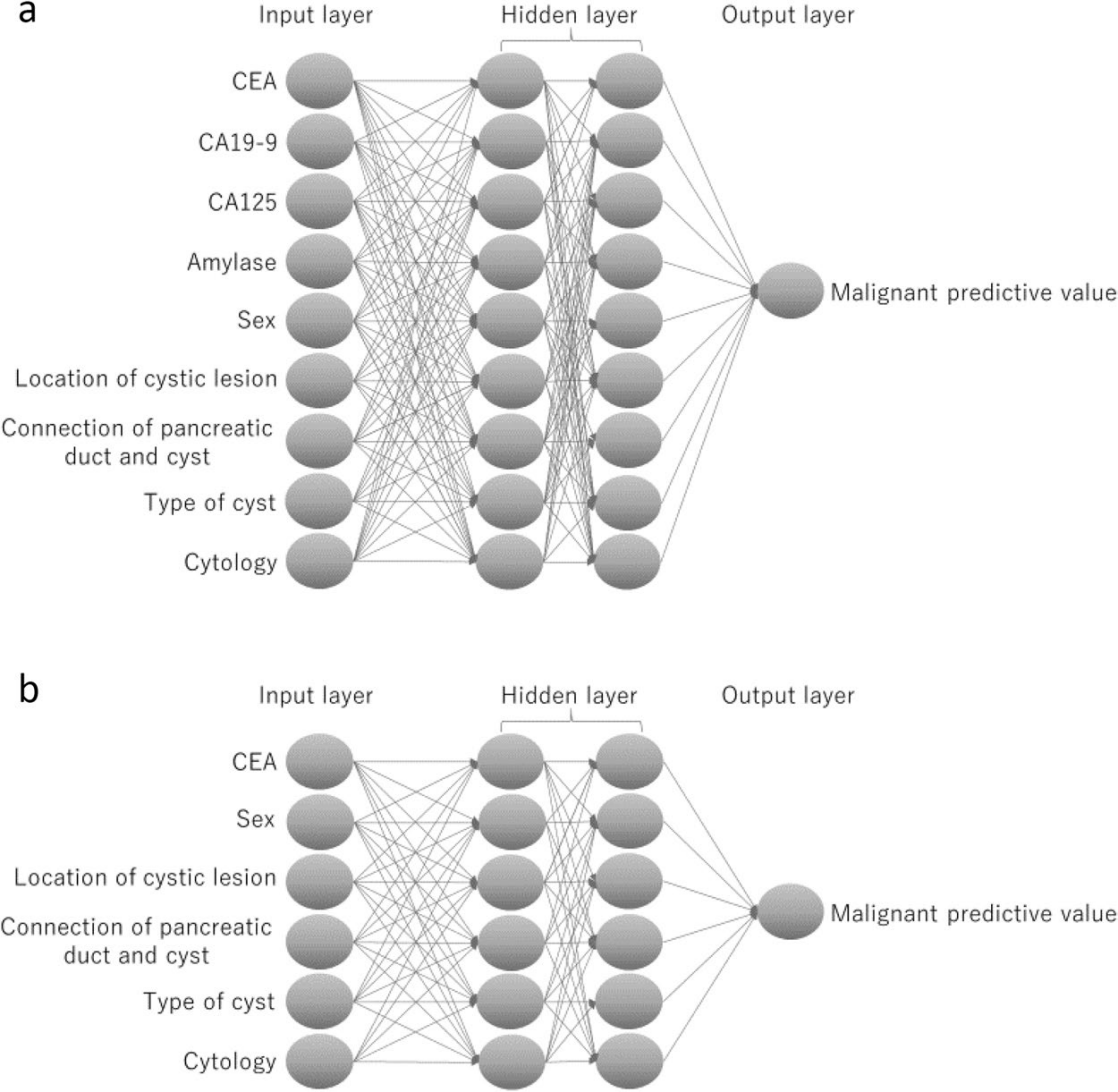
Algorithm of deep learning. Data are passed from layer to layer: from the input layer to the output layer. (**a**) Algorithm of artificial intelligence (AI). (**b**) Algorithm of AI using only carcinoembryonic antigen (CEA) except carbohydrate antigen (CA) 19-9, CA125 and amylase.

A neural network is a machine learning algorithm that provides mathematical result after entering various numerical values or information. In this study, to construct an AI-based diagnostic algorithm, deep learning, a multi-hidden layer of neural network, was used (Fig. 3a). TensorFlow version 1.5 (Google LCC, Mountain View, USA) was used for the deep learning analysis. During the training process of deep learning, labelled information such as clinical information was entered into the deep learning algorithm. Subsequently, output values were calculated. Data were labelled as follows: malignant cystic lesion was defined as 1 and benign as 0 according to the final diagnosis. In this study, the input layers were CEA, CA19-9, CA125, amylase in the cyst fluid, sex, cyst location, connection of the pancreatic duct and cyst, type of cyst (monolocular or multilocular), and cytology. The value of the input layer was normalised and keyed in the algorithm. The hidden layer has two layers, and each hidden layer has nine nodes. Tanh (hyperbolic tangent) was used for activation function of the hidden layer. The softmax function was used for the activation function of the output layer. The optimisation algorithm used to train the network weights was stochastic gradient descent. The output layer was defined as malignant predictive value calculated by AI using deep learning. The malignant predictive value is a continuous variable from 0 to 1. When the malignant predictive value is close to 1, the diagnosis of AI using deep learning was regarded as malignant cystic lesions. The accuracy validation method used fivefold cross validation: (learning/test sample ratio: 80%/20% × 5). In other words, 80% of all cases were randomly assigned to the learning sample and 20% to the test sample, and its diagnostic ability was measured five times. In addition, because none of the markers are routinely used or recommended in the fluid analysis other than CEA in clinical practice, we also created AI algorithm using only CEA and clinical data (sex, cyst location, connection of the pancreatic duct and cyst, type of cyst and cytology) except CA19-9 CA125 and amylase (Fig. 3b).

### Endpoint

The primary endpoint was to investigate and compare the diagnostic ability of AI, AI using only CEA, cyst fluid analysis (CEA, CA19-9 CA125, and amylase) and cytology in differentiating malignant from benign pancreatic cystic lesions. The main outcomes were diagnostic sensitivity, specificity, PPV, NPV, and accuracy.

The secondary endpoint was factors of malignant pancreatic cystic lesion. Factors of malignant pancreatic cystic lesion were analysed using univariate and multivariate analyses. Variables employed for analyses were CEA, CA19-9 CA125, amylase, sex, location of the cystic lesion, connection of the pancreatic duct and cyst, type of cyst, cytology, and AI.

### Statistics

Chi-squared test (or Fisher’s exact test, if appropriate) was used for categorical variables and Mann-Whitney U test used for continuous variables in the univariate analyses. McNemar test was used to calculate the sensitivity, specificity, and accuracy. A P value < 0.05 was considered significant. ROC curve analysis was performed to characterise the diagnostic ability of cyst fluid tumour markers, amylase, cytology, and AI in differentiating malignant from benign cysts. The AUC was calculated to define the cut-off value for the diagnosis of malignant cystic lesions. The value at the top left was considered the cut-off value by ROC. Logistic regression models for multivariate analysis were used. Variables with p < 0.05 in the univariate analysis were selected for entry into the multivariate analysis. These statistical analyses were performed using SPSS version 21 software (IBM, Armonk, NY, USA).
